# A Rare Case of Carpal Osteomyelitis in a Spinal Cord Injury Patient: A Case Report

**DOI:** 10.7759/cureus.36283

**Published:** 2023-03-17

**Authors:** Royce Copeland, Erica Blanchard, Paige Saito

**Affiliations:** 1 Physical Medicine and Rehabilitation, Baylor College of Medicine, Houston, USA; 2 Physical Medicine and Rehabilitation, University of Pennsylvania, Philadelphia, USA; 3 Medicine, Touro University California, Vallejo, USA

**Keywords:** scaphoid, acute inpatient rehabilitation, carpal bones, spinal cord injury, osteomyelitis

## Abstract

Osteomyelitis of the carpal bones is rare and usually occurs in the setting of penetrating trauma. Here, to our knowledge, we report the first known documented case of carpal osteomyelitis in a spinal cord injury (SCI) patient and discuss the medical management of this patient. A 62-year-old male with a remote history of traumatic SCI at T5 American Spinal Injury Association (ASIA) Impairment Scale (AIS) A and a history of IV polysubstance abuse presented to an acute care hospital for acute non-traumatic right dorsal wrist pain. Initial hand and wrist X-rays were negative for acute findings. After eight weeks of continued symptoms, severely impaired activities of daily living, and decreased independence, the patient was admitted to acute rehabilitation. MRI showed bone edema changes involving the distal radius, scaphoid, lunate, majority of the capitate, and hamate, concerning possible osteomyelitis. A CT-guided biopsy of the scaphoid confirmed methicillin-resistant Staphylococcus aureus (MRSA) osteomyelitis. He completed a seven-day course of IV vancomycin followed by 12 weeks of oral doxycycline. A follow-up positron emission tomography (PET) scan showed no evidence of osteomyelitis, and the patient returned to a baseline functional status of modified independence for most activities of daily living. Carpal osteomyelitis in SCI patients is rare and can be challenging to diagnose, given that it can present with a lack of systemic symptoms and nonspecific laboratory markers. This is the first documented case of carpal osteomyelitis involving an SCI individual. The continuation of diminishing hand mobility, function, and independence should prompt further workup with MRI to rule out uncommon but potentially debilitating diseases such as osteomyelitis.

## Introduction

Infections are a significant cause of morbidity and mortality in patients with spinal cord injury (SCI). UTIs, pneumonia, skin/soft tissue infections, and osteomyelitis are the most common infections among this population. SCI individuals are at high risk for community-acquired and hospital-associated infections due to frequent contact with healthcare systems, chronic use of medical devices, decreased physical activity, and a predisposition to being immunocompromised [1]. Although not the most common infection in the SCI population, osteomyelitis can frequently develop from an extension of infection from pressure ulcers. The individual’s inability to change position due to impaired motor function or decreased sensation leads to reduced tissue perfusion, thus increasing the patient’s susceptibility to developing pressure ulcers [2]. The most common source of osteomyelitis in SCI patients is a local infection from pressure ulcers at bony prominences, which include sacrum, ischial tuberosity, trochanteric area, and malleoli [1,2]. The localization of osteomyelitis involving the hand or wrist is uncommon in the general population, especially in the absence of trauma or other sites of boney infection [3]. However, when it occurs, osteomyelitis of the hand is most commonly seen in the phalanges or metacarpals. It is usually the result of direct contamination from penetration injuries, open fractures, or contiguous spread from sites of infection [3,4]. The authors of this case report present the first documented case of carpal osteomyelitis involving an SCI patient and discuss the treatment option for this patient along with their functional outcomes.

## Case presentation

**Figure 1:**
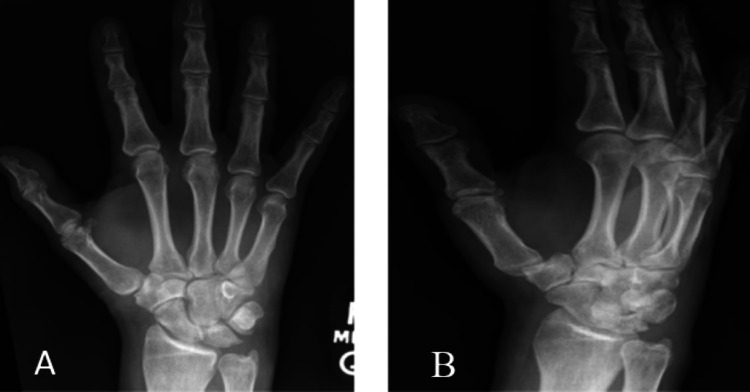
(A) Frontal and (B) lateral view. X-ray images of the hand at the initial presentation.

A 62-year-old right-hand dominant male with a significant history of type two diabetes mellitus, traumatic SCI T5 American Spinal Injury Association (ASIA) Impairment Scale (AIS) A secondary to a work-related fall in 2019 and a history of IV polysubstance abuse prior to SCI, presented to an acute care hospital for acute non-traumatic right dorsal wrist pain. Initial workup was significant for elevated C-reactive protein (CRP) of 21.89; however, uric acid, erythrocyte sedimentation rate (25), WBC, antinuclear antibodies, and creatine kinase were within normal limits. Initial right-hand and wrist X-rays showed no acute osseous abnormality (Figure 1). The patient was discharged from his acute hospitalization with conservative therapy, including non-steroidal anti-inflammatory medications and physical therapy.

**Figure 2:**
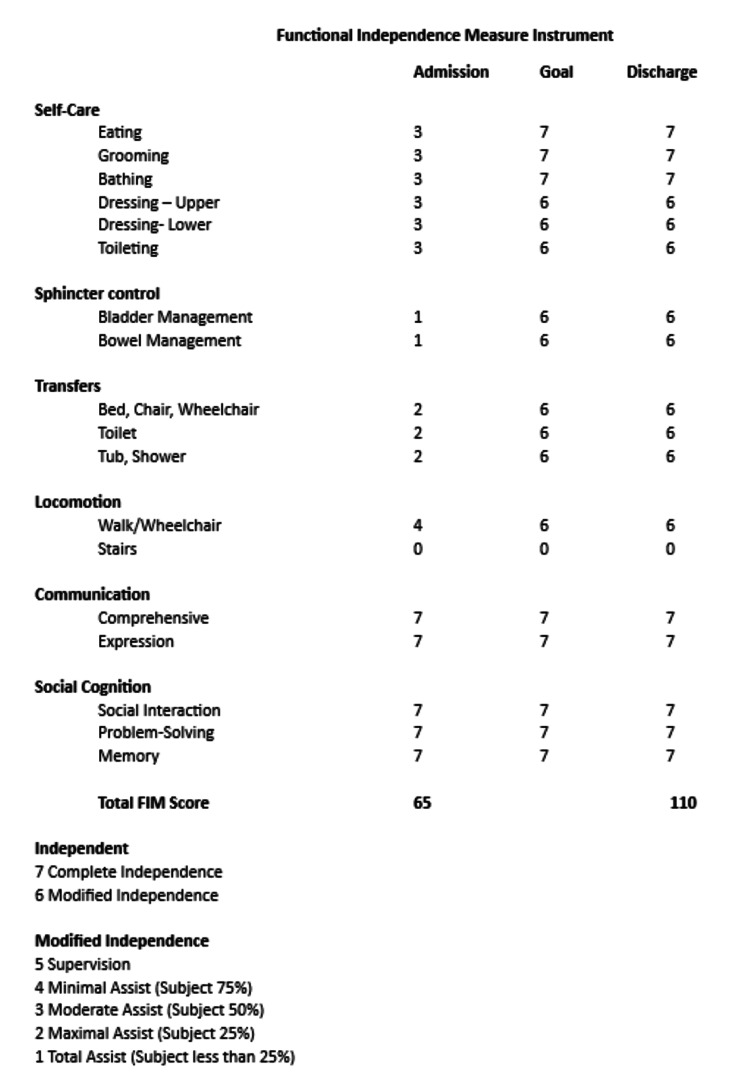
General Functional Independence Measure. General functional independence measure at admission and discharge of the patient.

After eight weeks of worsening symptoms of pain, decreased mobility, and impaired function, the patient was admitted to veterans affairs acute inpatient rehabilitation for severe impairments in activities of daily living. During the interview, he denied any recent trauma, IV drug use, fevers, chills, night sweats, or general fatigue. The primary symptoms included severe pain and stiffness at rest and during activities. Vital signs were within normal limits. The physical examination showed no skin changes or deformities and was positive for point tenderness at the right anatomic snuffbox and limited passive and active range of motion with flexion, extension, abduction, and adduction secondary to pain. Sensation to light touch was intact. His functional status prior to the hand injury was modified independence with most activities of daily living. However, his painful condition has made transfers difficult due to pain with wrist extension and inability to perform self-bladder and bowel management. The current functional status on admission was moderate to maximum assistance with most activities of daily living, with a general (Functional Independence Measure [FIM]) of 65 (Figure 2).

**Figure 3:**
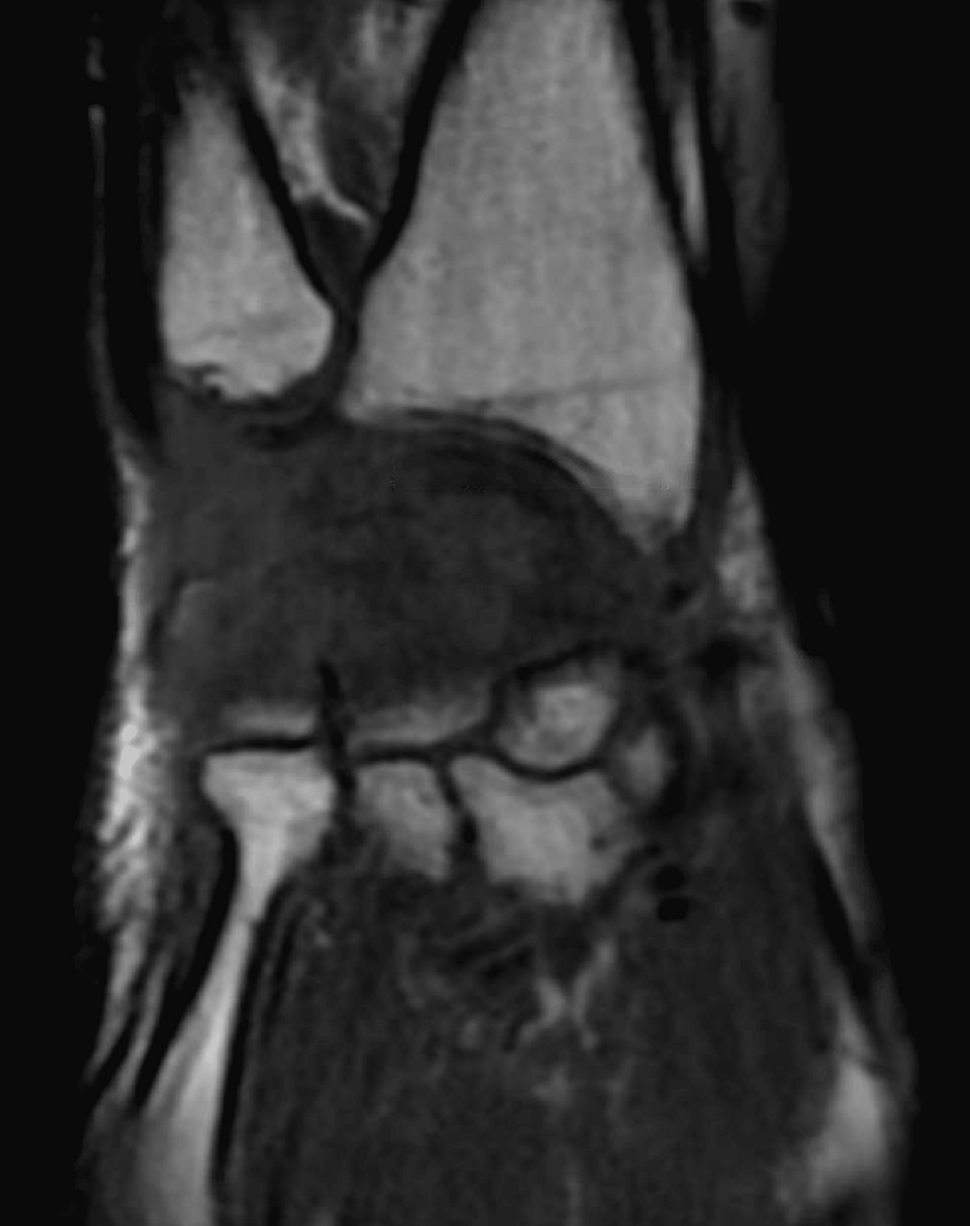
Coronal T1 non-fat suppressed sequence MRI. The right wrist demonstrates significant bone marrow edema throughout the scaphoid, lunate, majority of the capitate, and hamate with multifocal osseous erosions within the proximal and distal pole of the scaphoid. Findings concerning osteomyelitis and recommend follow-up with a bone biopsy.

Due to persistent pain, further imaging with MRI with a contrast of the hand and wrist was performed. It showed significant bone marrow edema changes involving the distal radius, scaphoid, lunate, majority of the capitate and hamate, and to a lesser extent involving portions of the proximal trapezium and trapezoid with osseous erosions within the proximal and distal poles of the scaphoid (Figure 3). Inflammatory markers were collected, resulting in a CRP level of 5.80 and an ESR level of 44. The patient underwent a CT-guided biopsy of the scaphoid, resulting in positive bone cultures for methicillin-resistant Staphylococcus aureus (MRSA) osteomyelitis. He completed a seven-day course of IV vancomycin, 1500 milligrams every 12 hours, followed by 100 milligrams of oral doxycycline twice daily for twelve weeks. Infectious disease consultation recommended positron emission tomography (PET) with CT after completion of antibiotics, and results showed no residual signs of osteomyelitis. Inflammatory markers were collected at discharge, with a CRP level of 0.60 and an ESR level of 4. No adverse effects occurred during his antibiotic course. The patient was discharged home at baseline functional status with modified independence of most activities of daily living and a general FIM score of 110. 

## Discussion

Osteomyelitis of the hand and wrist represents 1-6% of all upper extremity infections and only 10% of all cases of osteomyelitis [5]. Carpal osteomyelitis is often challenging to diagnose and manage, given its rarity, lack of systemic symptoms, and nonspecific laboratory markers. SCI patients are at high risk for osteomyelitis from direct inoculation of bacterial infections at the site of pressure ulcers. This often occurs near areas of bony prominences, and when necrotic tissue is not removed, this can lead to deeper bacterial penetration with surrounding tissue being infected [6]. Osteomyelitis of the hand and wrist are often approached with lower suspicion in cases of hand pain, especially in the absence of trauma. As a result, hand/wrist osteomyelitis is often diagnosed dissonantly from symptom onset. This patient presented with similar presentations as other reported hand/wrist osteomyelitis cases, including localized pain and stiffness without fever, chills, malaise, and night sweats but consistently elevated CRP [4]. While initial radiographic studies may show no acute changes, subsequent radiographs may be required as there is often a delay in the visualization of lytic lesions despite the progression of infection [5,7]. If there is a high clinical suspicion of osteomyelitis, MRI provides high sensitivity for detecting osteomyelitis, approximately 90%, but lower specificity of 70-80% [8]. Bone marrow edema is a common sign of osteomyelitis on T1-weighted images [8]. However, a bone biopsy is the gold standard of diagnosis. Further workup with bone cultures should be obtained to tailor therapy to treat the pathologic organism and rule out other pathologies [5,8,9]. 

With complete paraplegia and subsequent dependence on hand function, our case highlights the importance of having osteomyelitis as a potential differential diagnosis related to hand and wrist pain. Paraplegic patients depend on their hands and upper limbs to perform everyday tasks such as dressing, feeding, transfers, and transporting, all of which are important to achieve greater independence. The patient's history of complete SCI, diabetes mellitus, and IV drug use put this individual at significant risk for osteomyelitis as a potential source of his hand pain. Immunocompromised individuals, such as those with a history of diabetes mellitus and IV drug use, are at risk for hand infections due to a combination of leukocyte dysfunction and affected wound healing [5]. While laboratory studies such as ESR, CRP, and WBC can aid in diagnosing osteomyelitis, their findings are generally nonspecific and normal in over 50% of patients with osteomyelitis [5,7]. Although the patient denied current drug use and refused drug testing, the authors hypothesize this individual with a history of IV drug use possibly introduced harmful bacteria at the wrist with needles months prior to the onset of this painful condition leading to his MRSA osteomyelitis, as there was no reported trauma involved in this patient's case or prior bacteremia hospitalizations that could lead to a plausible explanation for this rare occurrence. The treatment of osteomyelitis located at the carpal bones is controversial, and recommended treatments are based on patient clinical stability and clinician experience. Surgical techniques include debridement, resection, or amputation of the infected bone in conjunction with oral or IV antibiotics [10]. In this case, the involvement of numerous carpal bones, clinical stability, and projected recovery process for an individual largely dependent on their hand function made the surgical options less desirable for this situation. Several studies concluded that antibiotics alone might be adequate and surgery should be reserved for aggressive lesions or osteomyelitis refractory to antibiotics [10]. In this case report, the patient was satisfied with the outcome utilizing only antibiotics and no surgical intervention. The projected recovery process of a corpectomy and dependence on hand function ultimately left the patient opting for a prolonged antibiotic course over surgery.

## Conclusions

In our case report, carpal osteomyelitis in a complete SCI patient was adequately treated with one week of IV vancomycin and 12 weeks of oral doxycycline. A PET scan showed the resolution of the infection, and the patient returned to his baseline functional status. Although this case demonstrates the efficacy of prolonged antibiotic treatment in a complete paraplegic SCI patient who developed carpal osteomyelitis, it is limited being a single retrospective study. More individual reports and prospective controlled studies need to look at the functional outcomes following treatment of hand or wrist osteomyelitis with antibiotics vs. surgery in these SCI patients or other comorbidities dependent on hand function for mobility and independence. Osteomyelitis is often not the first differential for patients with a chief complaint of hand or wrist pain. However, given the importance of hand function for SCI patients, it is essential for physicians to be aware of this diagnosis and know that antimicrobial therapy without surgical intervention is a viable option for this population.

## References

[1] Garcia-Arguello LY, O'Horo JC, Farrell A, Blakney R, Sohail MR, Evans CT, Safdar N (2017). Infections in the spinal cord-injured population: a systematic review. Spinal Cord.

[2] Abbasi F, Soolmaz K (2018). Infectious complications after spinal cord injury. Essentials of Spinal Cord Injury Medicine.

[3] McDonald LS, Bavaro MF, Hofmeister EP, Kroonen LT (2011). Hand infections. J Hand Surg Am.

[4] Burns J, Moore E, Maus J, Rinker B (2019). Delayed idiopathic hardware-associated osteomyelitis of the scaphoid. J Hand Surg Am.

[5] Pinder R, Barlow G (2016). Osteomyelitis of the hand. J Hand Surg Eur Vol.

[6] Vecin NM, Gater DR (2022). Pressure injuries and management after spinal cord injury. J Pers Med.

[7] Lee YJ, Sadigh S, Mankad K, Kapse N, Rajeswaran G (2016). The imaging of osteomyelitis. Quant Imaging Med Surg.

[8] Maffulli N, Papalia R, Zampogna B, Torre G, Albo E, Denaro V (2016). The management of osteomyelitis in the adult. Surgeon.

[9] Dhar SA, Mir MR, Butt MF, Kawoosa A (2007). Chronic osteomyelitis of the carpal trapezoid. J Hand Surg Eur Vol.

[10] Sendi P, Kaempfen A, Uçkay I, Meier R (2020). Bone and joint infections of the hand. Clin Microbiol Infect.

